# Evaluation of WASPLab Software To Automatically Read chromID CPS Elite Agar for Reporting of Urine Cultures

**DOI:** 10.1128/JCM.00540-19

**Published:** 2019-12-23

**Authors:** Matthew L. Faron, Blake W. Buchan, Hasan Samra, Nathan A. Ledeboer

**Affiliations:** a Medical College of Wisconsin, Milwaukee, Wisconsin, USA; b Wisconsin Diagnostic Laboratories, Milwaukee, Wisconsin, USA; Washington University School of Medicine

**Keywords:** automation, chromogenic media, urinary tract infection

## Abstract

Urine cultures are among the most common specimens received by clinical laboratories and generate a major share of the laboratory workload. Chromogenic agar can expedite culture results, but technologist review is still needed. In this study, we evaluated the ability of the WASPLab software to interpret urine specimens plated onto chromID CPS Elite (CPSE) agar. Urine specimens submitted for bacterial culture were plated onto CPSE agar with a 1-μl loop using the WASP. Each plate was imaged after 0 and 18 h of incubation, and colonies were enumerated by color using the WASPLab software and a technologist’s reading from a high-definition (HD) monitor. The results were reported as negative if <10 colonies/plate were detected. Laboratory information system (LIS) time stamps were used to measure the time to result. A total of 1,581 urine cultures were tested. The sensitivity and specificity of the software were 99.8% and 68.5%, respectively, which included 2 manual-positive/automation-negative (MP/AN) results and 170 manual-negative/automation-positive (MN/AP) results. Of the 170 MN/AP specimens, 116 were caused by microcolonies missed by the technologist. The remaining MN/AP results were caused by either count differences near the 10-colony threshold (*n* = 43) or count differences of >50 CFU (*n* = 11). The use of both CPSE agar and the WASPLab software improved the time to result for urine culture, reducing the average time to result by 4 h 42 min for negative specimens and 3 h 28 min for positive specimens compared to that with standard-of-care testing. These data demonstrate that the use of CPSE agar and automated plate reading has the potential to improve turnaround time while maintaining high sensitivity and reducing urine culture workload.

## Abstract (precis)

Urine cultures are among the most common specimens received by clinical laboratories and generate a major share of the laboratory workload. Chromogenic agar can expedite culture results, but technologist review is still needed. In this study, we evaluated the ability of the WASPLab software to interpret urine specimens plated onto chromID CPS Elite (CPSE) agar. Urine specimens submitted for bacterial culture were plated onto CPSE agar with a 1-μl loop using the WASP.

## INTRODUCTION

Urinary tract infections (UTIs) are some of the most common bacterial infections, with estimates of up to 150 million cases a year worldwide (1, 2). In the United States alone, UTI symptoms account for almost 1% of all clinic visits, and the societal costs associated with these infections are calculated to cost the United States approximately $3.5 billion a year based on health care costs and lost productivity (3, 4). The high occurrence of UTIs and the ease of specimen collection create a large burden on clinical laboratories, accounting for 24 to 80% of all cultures set up, depending upon the laboratory’s serviced population (5).

Laboratory diagnosis of UTIs is based on urinalysis, including several markers such as nitrite, leukocyte esterase, and microscopic urinalysis white blood cell count; however, bacterial culture is necessary for definitive diagnosis of symptomatic and asymptomatic UTIs (6, 7). Interpretation of bacterial culture is dependent upon the bacterial burden, diversity, and clinical scenario, including assignment of uncomplicated versus complicated infection. Complicated UTIs are typically defined as a patient with a structural or functional urinary tract abnormality or altered immune system, whereas uncomplicated UTIs are characterized as females of childbearing age with symptoms of dysuria or urgency but lacking systemic symptoms (8). For uncomplicated UTIs, current Infectious Diseases Society of America (IDSA) guidelines define a bacterial burden of ≥10^4^ CFU/ml as significant (9). This threshold is reduced to 10^3^ CFU/ml when specimens are tested from patients with complicated UTIs due to their increased risk and likelihood of disseminated or severe infection (10).

Bacterial burden is a key factor for interpretation; however, accurate interpretation and diagnosis require differentiation of active infections versus contamination or colonization. The majority of uncomplicated UTIs are caused by a single pathogen, so many laboratory guidelines recommend against full identification and reporting of cultures containing 3 or more possible pathogens at >10^4^ CFU/ml, as these are indicative of poor-quality specimens (5, 8, 11). Furthermore, specimens that contain a majority of skin and genital flora should also be disregarded as poor specimen collections. The most common UTI pathogens include Escherichia coli, Klebsiella pneumoniae, Enterococcus spp., Proteus mirabilis, and Pseudomonas aeruginosa (12).

Due to the high volume of cultures and technical expertise needed to properly report urine cultures, selective and chromogenic media have been developed to improve workflow and reduce overall cost. Several chromogenic agars are FDA cleared and commercially available to aid in the detection of potential uropathogens, and studies comparing these media to traditional culture with blood and MacConkey agar have found equivalent sensitivity (13). Automation in the clinical laboratory has developed significantly over the years, beginning with automated streaking instrumentation and culminating in total laboratory automation (TLA) that inoculates, streaks, images plates, and incubates cultures. The two most prominent automation systems are the WASPLab software (Copan Diagnostics, Brescia, Italy) and the BD Kiestra system (Becton, Dickinson, Franklin Lakes, NJ), both of which have been reported to improve consistency in plating and free up the technologist’s time to perform other duties (14, 15). Image analysis software has been added to these systems and has demonstrated high sensitivity for detecting growth on chromogenic agar for patient screening (16–18).

In this study, the performance of the WASPLab chromogenic detection module (CDM) software to aid in the initial segregation of negative versus nonnegative urine cultures using chromID CPS Elite (CPSE) agar (bioMérieux, Marcy l’Etoile, France) was evaluated. CPSE agar is a chromogenic agar designed specifically for use with urine sample cultures that has the capability to directly identify E. coli and presumptively identify Enterococcus spp., *Enterobacteriaceae*, and members of the *Proteeae* (now *Morganellaceae*) group based on the use of different chromogens. Images of urine specimens plated to CPSE agar were analyzed using the CDM algorithm and compared to a technologist’s interpretation as the gold standard. Performance of bacterial counts, color, and turnaround time (TAT) was compared between manual and software reporting.

## MATERIALS AND METHODS

### Specimen processing.

Enrollment of urine specimens occurred from January 2017 through April 2017 at Wisconsin Diagnostic Laboratories, which services a primarily adult population for Froedtert Hospital and the surrounding clinics and long-term-care facilities. All testing was performed on clean-catch specimens from both in- and outpatients who fit the laboratory policy for bacterial culture (<24 h for unpreserved and <72 h preserved). These specimens included both unpreserved and preserved (in boric acid) collections. All plating was performed by the WASP using a 1-μl loop, and plates were transferred to the WASPLab software for incubation and imaging. Testing was performed in parallel with standard-of-care (SOC) testing.

### Manual scoring of chromogenic plates and urine culture.

Multiple technologists were used in the study, and each was trained on how to read CPSE agar prior to the initiation. In addition, a reading guide was placed next to the bench for quick access when reporting results. All testing was integrated into the SOC testing, which followed the laboratories’ standard practices. Briefly, urine cultures imaged at 18 h are populated into a WASPLab workstation list. Technologists view all images on an HD monitor, preliminarily report negative cultures, and pull any plates that need further workup. In this study, the technologist read both blood agar plate (BAP) and MacConkey (MAC) agar cultures as well as the CPSE agar cultures. Specimens are worked up 24 hours a day, 7 days a week and have two technologists during first shift (06:30 to 15:30). During second and third shifts, a single technologist performs the preliminary image review and follow-up work. Results are recorded into the LIS, and plates that need further workup are sent to a stack on the WASPLab. In batches, technologists work up these cultures, which include creating purity plates, performing matrix-assisted laser desorption ionization (MALDI), and setting up antimicrobial susceptibility testing (AST) (if possible). No additional workup was required for CPSE agar after colony counts were recorded (image review). Culture results as well as the time to reporting for preliminary and final MALDI identification (ID) were obtained from the LIS time stamp. Final SOC results included laboratory expert rules to differentiate between poor collection and contamination. The expert rules used in this study were “multiple pathogens detected” and “growth of normal genital flora.” “Multiple pathogens detected” was defined as the identification of 3 or more Gram-negative organisms. “Growth of normal genital flora” was defined as growth over the 10-CFU threshold, but with the flora containing skin contaminants such as S. epidermidis or Corynebacterium spp. All technologists were blind to the software results.

### Digital analysis of chromogenic media.

Two images were taken of the media, one immediately after inoculation (time point 0) and then again after 18 h of incubation. Using the CDM image analysis software, each paired image was reviewed as previously published with some differences to the algorithm (17). In this revised version of the software, multiple hue, saturation, and value (HSV) color ranges were incorporated to differentiate between E. coli (red to burgundy), *Proteeae* (now *Morganellaceae*) (light brown to dark brown), *Enterococcus* spp. (turquoise), and KESC (*Klebsiella* spp., *Enterobacter* spp., Serratia spp., and Citrobacter spp.) group (green to blue green). Furthermore, the software analyzed the pixel differences used to define and count individual colonies to report the number of each organism present. Analysis was performed automatically after the 18-h image was taken, and the results, including time read and colony count based on color, were recorded. The software also reported counts of microcolonies, which are defined as colonies with a diameter of ≤0.2 mm.

### Discrepant analysis.

The software results were compiled and sent to the laboratory at the end of the study. This limitation required all discrepant analysis to be performed with only plate images, as all cultures had been discarded. Images of discordant specimens were sent to the laboratory for a second review. Discrepancies in the data were classified in three distinct categories, which included growth of microcolonies not detected by the technologist, count difference near the limit of significance, and count difference of ≥50 colonies. As the specimens were discarded, confirmatory identification was not possible.

### Statistical analysis.

The software reported colony count (based on chromogen color), and time analysis was completed, which were compared to manual reads as the gold standard. Sensitivity and specificity were calculated using standard methods and McNemar’s test (19). These calculations were performed for both growth/no growth and detection of colonies at the threshold level. Ninety-five percent confidence intervals (CIs) were calculated by using the efficiency score method (20). Statistical significance for time to result was performed using an unpaired 2-tailed Student's *t* test.

## RESULTS

### Specimen characterization and prevalence.

A total of 1,581 specimens were evaluated that contained both a CPSE agar software result and a technologist result to allow for a complete comparison. Enrolled specimens consisted of approximately a 3/1 ratio for unpreserved and preserved urine samples. Of these 1,581 specimens, 566 (35.8%) specimens contained <10 colonies (382 specimens showed no growth) based on technologist reporting. Another 13.3% of specimens were considered insignificant due to multiple pathogens or normal genital flora detected. The remaining 50.9% of the specimens were considered significant for pathogens, according to the policies defined by the laboratory.

### Performance of the software to read CPSE plates.

Images read by the technologist and software were recorded as either positive (≥10,000 CFU/ml) or negative (<10,000 CFU/ml). Comparisons were made according to 4 categories, manual positive/automation positive (MP/AP), manual negative/automation negative (MN/AN), manual negative/automation positive (MN/AP), and manual positive/automation negative (MP/AN). The results for comparison consisted of 1,013 specimens that were MP/AP, 396 specimens that were MN/AN, 170 specimens that were MN/AP, and 2 specimens that were MP/AN (Table 1). These data resulted in a sensitivity of 99.8% and a specificity of 69.9%.

**TABLE 1:** Performance of WASPLab software compared to manual read for 10 or more colonies

Analysis	No. with result of^*a*^:				% (95% CI) for measure of:	
	MP/AP	MN/AN	MN/AP	MP/AN	Sensitivity	Specificity
Real-time results	1,013	396	170	2	99.8 (99–100)	69.9 (66–74)
Postdiscrepant analysis	1,013	485	54	2	99.8 (99–100)	90.0 (87–92)

a MP/AP, manual positive/automation positive; MN/AN, manual negative/automation positive; MN/AP, manual negative/automation positive; MP/AN, manual positive/automation negative. *n* = 1,581.

The 172 discrepant images were sent back to the laboratory for a second review to determine the reason for the discrepancy (Table 2). Causes of MN/AP specimens were observed to fall into 3 separate categories, as follows: growth of microcolonies (≤0.2-mm diameter), bacterial counts near the limit of significance (≥10 CFU), and count differences of >50 colonies. The majority of the MN/AP results were due to the presence of microcolonies, which occurred in 68.2% (116/170) of the MN/AP specimens. Differences in manual versus software counting near the significance limit occurred in 25.3% (43/170) MN/AP specimens, while specimens containing a >50-colony count difference occurred in 6.5% (11/170) of specimens. The only trend observed in these 11 specimens was the presence of colonies with no color, which may have been missed or not reported by the technologist. The software’s definition of microcolonies often required zooming in on images to detect them manually, which may have been why these were often missed. A version of the software that ignored these microcolonies was used to reanalyze the data set, which improved the specificity of the reading to 90.0%. However, further studies are needed to determine what organisms these microcolonies were and if they are important for patient care.

**TABLE 2:** Discrepant analysis of manual-negative/automation-positive specimens

Discrepancy category	No. of plates
Counting microcolonies	116
Counts near the limit of detection	43
Counts with a >50-CFU difference	11

**FIG 1:**
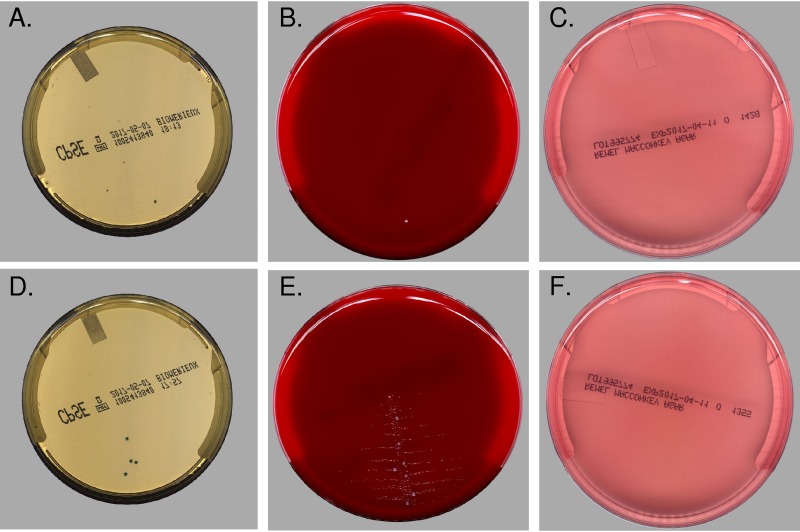
Images of the 2 manual-positive/automation-negative specimens. (A to C) The first specimen (in CPSE, BAP, and MAC agar, respectively) was manually reported as multiple organisms present with 1 pink colony and 100 no-color colonies. Automation reported out 6 colonies, with 2 pink, 2 no color, and 2 microcolonies. Visual reexamination of the images shows 2 pink colonies and 1 white colony. (D to F) The second specimen was manually reported as >100,000 Pseudomonas aeruginosa and 5,000 Gram-positive cocci in chains. Automation reported 2 microcolonies, 2 no-color colonies, and 4 turquoise colonies. The 4 turquoise colonies can be observed in the reexamination of the image, and 7 colonies are seen on the BAP agar (F). When enlarging both the CPSE and BAP plate images, a small dusting of possibly debris or discoloration can be detected, which can also be seen on the time 0 (T0) image (not shown). It is possible that the technologist misinterpreted this as growth. As plates were discarded by discordant analysis, confirmation cannot be confirmed, but the smaller growth is likely the P. aeruginosa, although it is unclear why no growth was observed on the MAC agar. It is possible that the poor growth on the BAP agar reduced the viability on the MAC plate.

**FIG 2:**
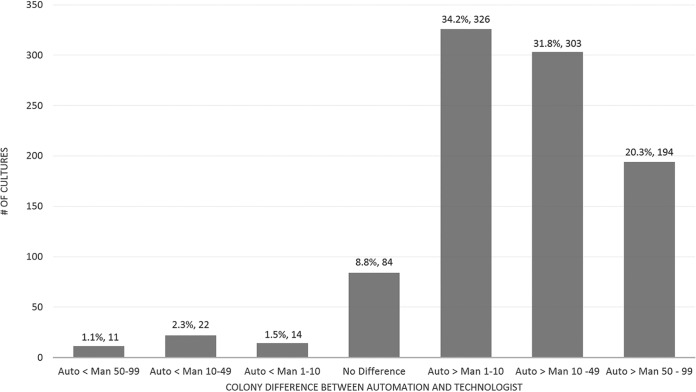
Evaluation of the software’s colony count accuracy for specimens containing 1 to 100 CFU. Ranges above 0 indicate higher automation counts. Data do not include specimens reported as >100,000 CFU/ml as the actual manual colony count is unknown. Auto, automated; Man, manual.

For the 2 MP/AN specimens, the software reported 5 and 6 colonies from the CPSE agar, and the technologist counted 102 and 104 colonies, respectively. Upon further review of the CPSE agar images, 3 and 4 colonies were observed on the two CPSE plates (Fig. 1A to C). The final report for one specimen stated that multiple organisms were present, which likely indicates contamination. The other specimen’s final report was >100,000 P. aeruginosa CFU with 5,000 CFU Gram-positive cocci in chains. It is unclear as to why the initial manual read for the CPSE agar reported greater than 100 colonies (Fig. 1D). Upon zooming in on the image on an HD monitor, small dust-like spots could be observed as either brown or colorless. These are also present on the T0 images. It is possible that technologists interpreted these as colonies and reported them as either no color or brown. Alternatively, the technologist could have reported from the blood agar plate that had small colonies at >100 CFU (Fig. 2E). A final possibility is that plates were recorded after removal from the incubator. Both specimens were reported 10 h after the images were taken, which could result in different growth not captured in the picture.

Colony counts were also performed, differentiating colony color by both the technologist and the software. Technologists counted only up to 99 CFU, and anything past this number was reported as >100 CFU. The software was not limited to a set amount and reported the total count on each plate. To evaluate the accuracy of the software to differentiate CFU, only plates that contained 1 to 99 colonies (technologist counts) were compared. The results were batched into 7 categories, which included no difference (0), colony count differences between 1 and 10 CFU (range, −10 to −1 and 1 to 10), differences in counts between 10 and 50 (range, −50 to −10 and 10 to 50), and count differences of >50 (range, −99 to −50 and 50 to 99) (Fig. 2). Results in the positive values indicate more colonies counted by the software, and counts reported as negative values indicate plates that had more colonies from the manual read. Plates containing >100 colonies were removed from these calculations, as it would not be an equal comparison due to the human limitations.

Comparing colony counts demonstrated the high accuracy of the software to differentiate colonies (Fig. 2). Out of 954 remaining specimens, 44.4% (424/954) fell within a difference of 10 or fewer CFU. In general, the software reported a higher number of colonies, at 823 colonies compared to 47 colonies with manual analysis. The trend in overcalling is expected, as a technologist will likely underestimate counts since the difference between 30 and 99 colonies is not clinically relevant and would have little effect on patient care.

Data analysis was also performed to determine the difference between growth and no growth on the plates. Growth was defined as any single colony, with microcolonies reporting by the software turned off, as they were undetectable without magnification by the technologist. The software and manual read results were concordant in 88.9% of specimens (1,406/1,581). Of the 175 discordant specimens, only 6 were manual growth/automation no growth. All but 1 of these had counts below 10 colonies. The remaining manual growth/automation no growth specimen had 15 colonies lacking pigmentation, and the final report was reported as multiple organisms present. Further review of this discordant specimen found that the software could detect 16 microcolonies. The majority of the automation growth/manual no growth specimens had fewer than 10 colonies on the plate (66.7%). The remaining discrepant specimens consisted of 18.7% with growth between 10 and 99 colonies and 14.6% that had bacterial counts of ≥100 colonies. Two trends appeared from the ≥100-colony discrepant specimens. One appears to be possible reporting errors by the technologist, as the final report had results that were not entered properly into the LIS. The other group was observed to have small light-blue colonies that were missed by the technologists. Based on the CPSE package insert, blue colonies fall into the KESC group, but no further definitions for small colonies are described.

### Evaluating time to result between conventional culture, CPSE agar, and software analysis.

Time of reporting was collected to determine the effects that the software and CPSE agar had on TAT. These readouts included the start of culture, defined as the first T0 image that was taken by the WASPLab, the time the software recorded a result, the time the technologist viewed the CPSE agar, and the time of identification (ID) from our SOC workup. The natural workflow of the laboratory was included as a part of the study design so that the normal functions of the laboratory, such as workload, phone calls, and other distractions, were considered. Time to reporting was calculated separately for positive and negative cultures.

**FIG 3:**
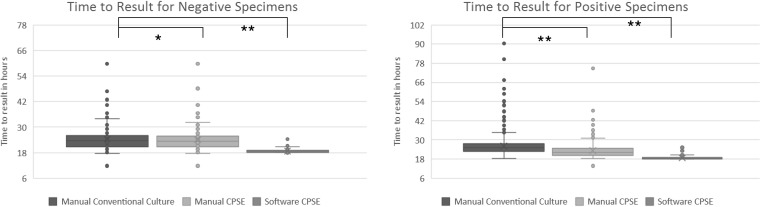
Distribution of time of result differences for negative and positive specimens between standard of care, manual CPSE, and software analysis. Significance was determined by a 2-tailed paired Student's *t* test (*, *P* = 0.046; **, *P* < 0.001).

There was a high variability in the TAT for manually read cultures, with results ranging from 18 h 23 min to 106 h 11 min. Results over 30 h were rare, and the majority clustered around 24 h from initial plating (Fig. 3). Conventional culture averaged a final time to result of 24 h 18 min for negative cultures and a final time to ID result of 26 h 8 min for positive cultures (Fig. 3). Manual reading of the CPSE reduced the TAT for negative specimens, which averaged 24 h 3 min (*P* < 0.05). Positive specimens using CPSE agar were reported on average faster than were negatives, at 23 h 15 min; however, these differences were likely caused by outliers that may have increased the average time to result in negative specimens. Time to result for positive specimens was significantly shorter than standard time to ID (*P* < 0.01), but this value would only be useful for E. coli-only cultures, as no workup is needed. The median TAT for conventional and CPSE agar were 23 h 33 min and 23 h 37 min for negative specimens and 25 h 7 min and 22 h 17 min for positive specimens.

Significant improvements in TAT were observed for positive and negative specimens in a comparison of software analysis to conventional testing and manual reading of CPSE agar (*P* < 0.01). Overall, the software had an average TAT of 18 h 44 min, with a median of 18 h 17 min when analyzing negative specimens and an average of 18 h 40 min and median of 18 h 18 min for positive specimens (Fig. 3). Compared to manual reporting of the CPSE agar, this resulted in a median reduction in TAT of 3 h 28 min for positive specimens and 4 h 42 min for negative specimens. The reduction in TAT was even greater in conventional culture, with a median reduction of 6 h 23 min for positive specimens and 4 h 48 min for negative specimen results. These calculations were performed using all positive specimens, but specimens positive only for E. coli would be possible to report using the software alone. In a comparison of the time of result for E. coli-only specimens, similar results were found, with a median difference of 3 h 1 min compared to CPSE agar and 6 h 28 min compared to SOC identification.

## DISCUSSION

The goal of both chromogenic agar and automation is to reduce the workload of the clinical microbiology laboratory and improve the TAT for patient results. Other studies have looked at the effects on TAT for CPSE agar using only manual reading and reporting. One study evaluated 200 urine specimens using the CPSE agar and found that the time to ID for E. coli was significantly reduced (2.7-h reduction) in a comparison of bacterial culture on blood agar and matrix-assisted laser desorption ionization–time of flight mass spectrometry (MALDI-TOF MS) for identification (21). The study also evaluated hands-on time and consumable usage and found that CPSE agar required 30 s less hands on time (on average) and used 1 less swab and biochemical test per specimen. Their data suggested that in a medium-size laboratory performing an average of 300 urine samples a day, over 900 technologist hours would be saved annually. Furthermore, the TAT differences observed in the Yarbrough et al. (21) study and the current study are likely the minimum expected differences due to the method of identification. Both studies used MALDI-TOF MS, which allows identification in minutes or in less than a few hours when batch testing is performed. As MALDI-TOF MS is among the quickest methods used for identification (spot biochemicals are quicker but not always used in labs with MALDI for final ID), laboratories that rely on slower automated ID systems, such as the Phoenix system (BD, Sparks, MD, USA), the Vitek 2 system (bioMérieux, Marcy l’Etoile, France), or MicroScan (Beckman Coulter, CA, USA), should expect a larger difference in TAT, as these assays require a minimum of 6 to 8 h for ID results.

The addition of software analysis did reduce the TAT over that of manual reading; however, there are some limitations to the data analysis that may skew results compared to the actual impact if implemented in the clinical laboratory. The time of result was calculated based on the time the software analysis was performed, but the requirement of CPSE agar to have additional workup for species identification of *Enterococcus* spp., KESC organisms, and *Proteeae* (now *Morganellaceae*) was not captured. In these cases, additional biochemical testing would need to be performed so the actual time to ID would be similar to the manual read. CPSE agar can directly identify E. coli colonies, so an 18-hour reporting could be achieved for both negative plates and pure E. coli cultures. In this study, automation-negative specimens accounted for 21.6% of all specimens, and cultures containing E. coli without other significant organisms accounted for 11.8% of the specimens. These cultures combined could allow the software to either presort or autoreport 33.4% of the urine cultures enrolled. An additional 7.3% of specimens could also be reported as significant for E. coli but contained other pathogens with potential clinical significance that would need additional workup. The remaining 59.3% of specimens that do not fall into these categories would require full workup by a technologist.

An unexpected finding during this study was the software’s detection of microcolonies not detected by the technologists. Unfortunately, analysis was performed after testing was completed, so there were no specimens or plates to go back to for identification of these microcolonies. Growth of these slow-growing organisms is likely due to the enhanced recovery of microorganisms using smart incubators. A recent study in 2018 evaluated recovery of organisms from urine culture pre- and postautomation (22). Interestingly, the use of smart incubators allowed for routine detection of Neisseria gonorrhoeae from blood agar plates more often than did standard incubation. Others have found similar results, demonstrating increased recovery of Gram-positive rods, such as Actinomyces spp. and Gardnerella vaginalis (23). It is possible that these microcolonies may include pathogens, so further study is warranted.

Comparisons between technologist and software reporting were performed based on specimens being positive when 10 or more colonies are identified; however, the software could differentiate between 9 different growth characteristics and was highly accurate in colony counts. Laboratory workflow could be improved by developing software rules that incorporate the laboratory’s standard-of-care practices. For instance, significant growth could be defined as one or two colony types being ≥10 colonies to remove the need for interpretation of plates that do not contain significant growth of a single colony (i.e., 5 Gram-negative rods [GNR] and 7 Gram-positive cocci [GPC]). In addition, any growth could be reported when source is defined as a catheter to follow IDSA guidelines. Poor collection could be identified by automation and segregated from positive cultures when 3 or more colony types are detected at ≥10 CFU/ml. Of the 1,581 specimens tested during this study, 401 (25.4%) specimens could have been determined to contain multiple organisms present using these additional rules. If validated along with reporting significant E. coli and negative cultures, these data suggest that 58.8% of all urine cultures could be reported at as early as 18 h postinoculation, which could greatly reduce the workload on a laboratory and allow physicians to make informed treatment decisions earlier.

Digital image analysis for the clinical microbiology laboratory continues to improve, but how it is integrated into the workflow will be dependent upon the laboratory leadership and internal validations. The software is highly flexible and can be set up to allow either minimal or moderate changes to the laboratory’s workflow. Implementation of the software could be used for rapid segregation of growth and no-growth cultures. Technicians could then batch view negative specimens and report the results in groups up to 40 specimens per click. This minimalistic adoption should allow technologists to focus their time on positive or complex specimens. Alternatively, the software could be integrated to maximize automation. In this approach, the software would automatically report negative cultures or cultures pure for E. coli infections. Technologists would then only view and work up positive urine cultures that were not pure for E. coli. E. coli-only cultures could also be sent to work up stacks for AST. In both cases, it would be vital to maintain a minimum of blood culture agar to identify group B streptococcus as well as some of the emerging pathogens, including Aerococcus spp. and Corynebacterium urealyticum (24). Finally, considerations would be needed for expert ruling of plate interpretation. Plates with multiple pathogens could be ruled as poor collection, which could either be confirmed by a technologist or autoreported. Overall, continued advancements to image analysis software should improve workflow, as has been observed for previous automation technologies.
